# The IDeaS initiative: pilot study to assess the impact of rare diseases on patients and healthcare systems

**DOI:** 10.1186/s13023-021-02061-3

**Published:** 2021-10-22

**Authors:** Ainslie Tisdale, Christine M. Cutillo, Ramaa Nathan, Pierantonio Russo, Bryan Laraway, Melissa Haendel, Douglas Nowak, Cindy Hasche, Chun-Hung Chan, Emily Griese, Hugh Dawkins, Oodaye Shukla, David A. Pearce, Joni L. Rutter, Anne R. Pariser

**Affiliations:** 1 Office of Rare Diseases Research (ORDR), National Center for Advancing Translational Sciences (NCATS), National Institutes of Health (NIH), Bethesda, MD 20817 USA; 2 NCATS NIH, Bethesda, MD 20817 USA; 3 Eversana Life Science Services, LLC, Chicago, IL USA; 4 Population Health Management Spring Hills MSO, Edison, NJ 08817 USA; 5 Oregon Health and Science University (OHSU), Portland, OR 97239 USA; 6 University of Colorado, Anschutz Medical Campus, Aurora, CO 80045 USA; 7 Sanford Health, Sioux Falls, SD 57117 USA; 8 Sanford Research, Sioux Falls, SD 57104 USA; 9 Sanford Health Plan, Sioux Falls, SD 57103 USA; 10 School of Medicine, The University of Notre Dame Australia, Sydney, Australia; 11 Sanford School of Medicine, University of South Dakota, Sioux Falls, SD 57105 USA

**Keywords:** Rare diseases, Health care costs, Diagnosis, Utilization

## Abstract

**Background:**

Rare diseases (RD) are a diverse collection of more than 7–10,000 different disorders, most of which affect a small number of people per disease. Because of their rarity and fragmentation of patients across thousands of different disorders, the medical needs of RD patients are not well recognized or quantified in healthcare systems (HCS).

**Methodology:**

We performed a pilot IDeaS study, where we attempted to quantify the number of RD patients and the direct medical costs of 14 representative RD within 4 different HCS databases and performed a preliminary analysis of the diagnostic journey for selected RD patients.

**Results:**

The overall findings were notable for: (1) RD patients are difficult to quantify in HCS using ICD coding search criteria, which likely results in under-counting and under-estimation of their true impact to HCS; (2) per patient direct medical costs of RD are high, estimated to be around three–fivefold higher than age-matched controls; and (3) preliminary evidence shows that diagnostic journeys are likely prolonged in many patients, and may result in progressive, irreversible, and costly complications of their disease

**Conclusions:**

The results of this small pilot suggest that RD have high medical burdens to patients and HCS, and collectively represent a major impact to the public health. Machine-learning strategies applied to HCS databases and medical records using sentinel disease and patient characteristics may hold promise for faster and more accurate diagnosis for many RD patients and should be explored to help address the high unmet medical needs of RD patients.

**Supplementary Information:**

The online version contains supplementary material available at 10.1186/s13023-021-02061-3.

## Introduction

When combined, rare diseases are not actually rare, as they collectively affect around 25–30 million people in the United States (US) and more than 300 million people worldwide [1–4]. RD represent a diverse spectrum of more than 7–10,000 different disorders, most of which affect only a few hundred to a few thousand people per disease [5–8]. It is estimated that around 85% of RD are genetic diseases, [6] the majority of which are serious or life-threatening conditions that carry substantial morbidity and early mortality, and present considerable medical and financial burdens to RD patients and the families who care for them [9–11]. Given the large number of different rare diseases, each of which affects only a small number of patients, assessing the true impact of rare diseases on healthcare systems (HCS) is challenging. RD are generally difficult to diagnose, with many patients undergoing prolonged diagnostic journeys, termed the diagnostic odyssey, in order to obtain an accurate diagnosis [12, 13]. Even when accurately diagnosed though, less than half of RD map to an International Classification of Disease (ICD) 10 code, with far fewer (< 20%) having a specific ICD 10 code, [14] resulting in most RD being under-recognized and under-counted within HCS databases (such as payor/insurance databases) [15–17] and myriad downstream effects, such as imprecise coding of RD patients and poor tracking and understanding of both RD patients and the diseases themselves. Further, without a diagnosis, it is often the case that a set of labs, notes, and other features (e.g., a computable phenotype) cannot be reliably or consistently used to identify RD patients. Hence, the true impact of RD on HCS are not well described, and RD remain largely invisible to the HCS.

There are some estimates in the medical literature that medical care for RD patients may account for more than 10% of overall costs in some HCS, [18] and a few small studies, mainly case series, have shown high direct medical costs of RD at single centers in individual diseases or narrow clusters of related diseases (e.g., severe/refractory seizures) [19, 20]. Recently, a patient-reported survey on direct and indirect costs of rare diseases in the U.S. was reported, which showed high direct and indirect medical cost burdens to patients and HCS, with total costs estimated to be about $1 trillion (US) in 2019 [21]. Another recent study examined pediatric and adult hospital discharges in patients with rare and common conditions, which showed substantially higher healthcare utilization in rare versus common diagnoses, with RD accounting for nearly half of the US national healthcare costs [22].

In order to better understand RD medical costs, more accurately identify RD patients, and shorten the diagnostic odyssey for RD patients, additional work needs to be done to develop generalizable methodologies and tools (e.g., clinical decision support tools) that can be used across different HCS to adequately and consistently identify RD patients within HCS and to objectively quantify direct medical costs associated with RD by disease and overall. Similarly, the impact of delayed or misdiagnosis of RD on patients and HCS has not been well quantified [13]. While delayed or misdiagnosis is an issue for both rare and common diseases, delays in diagnosis disproportionately impact RD patients given the often years-long diagnostic odyssey most patients undergo. Misdiagnosis and lack of diagnosis can result in inappropriate care, lack of targeted or, when available, disease modifying treatment, and missed opportunities for intervention that may ameliorate or prevent disease progression, which in some cases is irreversible or require administration within certain time windows (e.g., neurodegenerative or metabolic disorders) [23, 24].

IDeaS (Impact of Rare Diseases on Patients and Healthcare Systems) is a collaboration between the Office of Rare Diseases Research (ORDR) within the National Institutes of Health (NIH) National Center for Advancing Translational Sciences (NCATS), Eversana™, a commercial life sciences company, the Oregon Health & Science University (OHSU), Oregon’s public academic health center, Sanford Health (Sanford), a large integrated healthcare system predominately from the northern Midwestern states, and a health insurer in Australia. IDeaS is intended to be a small preliminary pilot study whose overall purpose is to explore the feasibility of identifying and describing RD patients in a limited set of 14 representative RD within different and diverse HCS. The 3 main aims are to: (1) explore whether methodologies could be developed to quantify patients with RD and provide estimates of disease prevalence in different HCS; (2) quantify the direct medical costs of a representative set of 14 RD in order to identify additional areas for study into RD direct costs and health burdens that may help identify gaps in RD research; and (3) perform a preliminary assessment of the diagnostic journey for selected patients in 2 RD (Batten disease [BD] late infantile neuronal ceroid lipofuscinosis type 2 [CLN2] and cystic fibrosis [CF]) to start to identify disease-course characteristics that might be used to inform the development of strategies that could accelerate RD diagnosis using graphical representation of the disease course in patient “journey maps” (Figs. 5, 6). While the IDeaS pilot study is limited in scope, it is hoped that the results of these explorations will contribute to further development of methods and approaches that can help us better understand the complex issues currently impeding our understanding of cost and utilization drivers for RD that could be applicable to the thousands of known RD, as well as to inform larger research questions, such as the relationships between costs and cost savings, patient outcomes and disease rarity. However, these lines of inquiry will require additional and iterative development of analytical tools and approaches that are beyond the scope of this study.

## Methodology

We conducted the IDeaS study, a retrospective, descriptive pilot study, to explore the feasibility of quantifying patient and direct medical costs for 14 representative RD (Table 1). There are 14 RD (or disease groups) included in the pilot that were selected by the study authors to explore a diverse set of disorders that differed in prevalence, organ systems affected, age of onset, clinical course, and availability of an approved treatment or specific ICD code, intended to be representative of many RD beyond the 14 used in this pilot. The pilot IDeaS study includes 3 main Aims for exploration.

**Table 1:** Rare diseases included in the IDeaS study, and affiliated ICD and CPT codes

Rare disease	Brief description	ICD code type (ICD-9 or 10)	ICD and CPT code(s)
Batten disease (BD) Also known as (aka) Neuronal Ceroid Lipofuscinoses (NCLs)	A group of genetic (inherited) disorders known as lysosomal storage disorders (LSDs) that result in progressive neurodegeneration due to accumulation of undigested cellular materials primarily in the Central Nervous System (CNS). BD is due to mutations in ~ 20 genes, with different types of BD having varying age of onset and disease progression. Clinical manifestations include seizures, cognitive and motor decline, and vision loss, among others	ICD-9	330.1 (cerebral lipidoses), 334.3
		ICD-10	E75.4 (neuronal ceroid lipofuscinosis), G11.1
Charcot Marie Tooth (CMT)	Group of inherited disorders where motor and/or sensory peripheral nerves degenerate over time, resulting in muscle weakness, wasting, and sensory loss, caused by abnormalities in the nerve axon or the myelin sheath around the long part of the nerve called the axon. Some signs and symptoms include muscle weakness, decreased muscle size, and decreased sensation, among others	ICD-9 And CPT	356.1 (Peroneal muscular atrophy) 81,324 (PMP22 (peripheral myelin protein 22) (eg; Charcot-Marie-Tooth; hereditary neuropathy with liability to pressure palsies) gene analysis; duplication/deletion analysis), 81,325, 81,326
		ICD-10 And CPT	G60.0 (Hereditary motor and sensory neuropathy) 81,324 [PMP22 (peripheral myelin protein 22)] (Duplication/Deletion DNA Test), 81,325 (PMP22), 81,326 (PMP22)
Cystic fibrosis (CF)	Progressive genetic disease due to mutations in the CFTR (cystic fibrosis transmembrane conductance regulator) gene. CF results in the accumulation of mucous in various cells and tissues which leads to persistent lung infections, nutritional problems, and other manifestations	ICD-9	277.00 (cystic fibrosis, without mention of meconium ileus), 277.0, 277.01, 277.02, 277.03, 277.09
		ICD-10	E84.9 (cystic fibrosis, unspecified), E84.0, E84.19, E84.11, E84.8
Eosinophilic esophagitis (EOE)	Chronic allergic inflammatory disorder of the digestive system characterized by large numbers of a certain white blood cell, the eosinophil, present in the esophagus. Common signs and symptoms include vomiting, stomach or chest pain, and difficulty swallowing or eating, among others	ICD-9	530.13 (eosinophilic esophagitis)
		ICD-10	K20.0 (eosinophilic esophagitis)
Focal and segmental glomerulosclerosis (FSGS)	Type of kidney disease that damages the filtering units (glomeruli) inside the kidney, resulting in scarring (sclerosis) and decrease of function within the kidney. Signs and symptoms can include swelling in body parts (e.g., legs), high blood pressure, loss of large amounts of protein in the urine, and high cholesterol, among others	ICD-9	581.1 (nephrotic syndrome with lesion of membranous glomerulonephritis), 581.2, 581.0, 581.3
		ICD-10	N04.1 (nephrotic syndrome with focal and segmental glomerular lesions), N04.0, N04.2, N04.7, N03.1
Hereditary hemorrhagic telangiectasia (HHT) aka Osler-Weber-Rendu disease	Inherited disorder that causes abnormal connections between arteries and veins, called arteriovenous malformations (AVMs), most commonly in the nose, lung, brain and liver. This can result in bleeding episodes such as nosebleeds, gastrointestinal (GI) tract bleeding (hemorrhaging), anemia, and strokes, among others	ICD-9	448.0 (hereditary hemorrhagic telangiectasia), 448.9
		ICD-10	I78.0 (hereditary hemorrhagic telangiectasia)
Lennox Gastaut syndrome (LGS)	A severe condition characterized by recurrent seizures (epilepsy) that begin early in life (typically between the ages of 3–5 years). Affected people have multiple types of seizures, changes in brain activity seen on electroencephalogram (EEG) and intellectual impairment	ICD-9	345.01 (generalized nonconvulsive epilepsy, with intractable epilepsy), 345.00, 345.0, 345.1
		ICD-10	G40.812 (Lennox Gastaut syndrome, not intractable, without status epilepticus), G40.811, G40.813, G40.814
Mitochondrial neurogastrointestinal encephalopathy (MNGIE)	A multisystem disorder characterized by progressive degeneration of the muscles of the GI tract and peripheral nerves, weakness of the eye muscles, arms and legs, and generalized wasting (cachexia), among others. MNGIE is one of the mitochondrial disorders, which are due to genetic mutations in the mitochondrial DNA. Mitochondria are present in virtually every cell in the body and generate most of the energy for a cell. Because energy is essential for tissues to function, mitochondrial diseases typically affect multiple organs in the body	ICD-9	277.87 (disorders of mitochondrial metabolism)
		ICD-10	E88.49 (other mitochondrial metabolism disorders)
Muscular dystrophy (MD)	A group of genetic diseases that damage or weaken muscles over time. There are several different types of MD that vary in the muscle groups affects, rates of progression, and signs and symptoms. Common signs and symptoms include trouble walking, breathing or swallowing, curvature of the spine and poor posture, drooping facial muscles or eyelids, among others	ICD-9	359.1 (hereditary progressive muscular dystrophy), 359.0, 359.21, 359.22, 359.23, 359.29, 359.9
		ICD-10	G71.0 (muscular dystrophy), G71.00 (muscular dystrophy, unspecified), G71.01 (Duchenne and Becker), G71.02, G71.09, G71.11
Osteogenesis imperfecta (OI)	Group of genetic diseases that mainly affect the bones, resulting in weak or malformed bones. OI is a group of several disorders that affect collagen, the underlying structural protein in bone, skin and other connective tissues, and vary in rates of severity and progression. Also referred to as “brittle bone disease”, OI can result in bones that break easily, often from mild trauma or from no apparent cause	ICD-9	756.51 (Osteogenesis Imperfecta)
		ICD-10	Q78.0 (Osteogenesis Imperfecta)
Pheochromocytoma (Pheo)	Rare type of tumor that usually arises from cells in the adrenal gland (chromaffic cells). The adrenals are located above each kidney. Pheo are usually benign, but cause the adrenal gland to make too many of certain hormones (e.g., catecholamines) that can lead to high blood pressure, and cause symptoms such as headaches and sweating	ICD-9 preceded by CPT	227.0 (benign neoplasm of adrenal gland) CPT: 82,384, 82,382, 83,835
		ICD-10 preceded by CPT	C74.10 (Malignant neoplasm of medulla of unspecified adrenal gland), C96.20, C96.29, D35.00 CPT: 82,382 (Catecholamines), 82,384 (Catecholamines, fractionated, 24-h urine without creatinine), 83,835 (Metanephrines, Fractionated, Free, LC/MS/MS, Plasma)
Sickle cell disease (SCD)	Inherited group of red blood cell disorders characterized by atypical hemoglobin molecules called hemoglobin S, which distort red blood cells into a sickle or crescent shape. Sickled cells can obstruct blood vessels leading to repeated episodes of pain (crises), infections, strokes, and bone/joint injury and among others	ICD-9	282.60 (sickle cell disease, unspecified), 282.41, 282.42, 282.61, 282.62, 282.63, 282.64, 282.68, 282.69, 282.6
		ICD-10	D57.1 (sickle cell disease without crisis), D57.20, D57.00, D57.01, D57.02, D57.211, D57.212, D57.219, D57.40, D57.412, D57.411, D57.419, D57.812, D57.811, D57.819, D57.80,
Takayasu’s arteritis (TA)	Rare type of vasculitis. Vasculitis is a group of disorders causing blood vessel inflammation. TA is a large vessel vasculitis that affects the aorta and its main branches, which carry blood from the heart to the rest of the body. Common signs and symptoms include arm or chest pain, high blood pressure, and narrowed, blocked or weakened arteries that can result in heart failure or stroke, among others	ICD-9	446.7 (takayasu’s disease)
		ICD-10	M31.4 (aortic arch syndrome—Takayasu)
Urea cycle disorder (UCD)	An inherited group of disorders that result in deficiency or absence of the activity of several enzymes (proteins) in the urea cycle, which affects how the body metabolizes (breaks down) amino acids (the building blocks of proteins). UCDs can result in the accumulation of ammonia, a toxic substance in the blood that can cause brain damage, coma and death	ICD-9	270.6 (disorders of urea cycle metabolism)
		ICD-10	E72.20 (disorder of urea cycle metabolism, unspecified), E72.2, E72.21, E72.22, E72.23, E72.29, E72.4

### Aim 1: Estimation of disease prevalence in different HCS databases

We initially attempted to identify patients with the 14 pilot RD within the 5 different HCS databases using diagnostic (ICD) codes (see Table 1, Additional file 1: Table S1); however, due to the substantially different billing methods used by the Australian healthcare system (see below), we were not able to reliably connect the Australian HCS data to the 14 RD used in the pilot. Thus, exploration and comparison of the Australian data could not be performed and was dropped from further consideration.

For the remaining 4 HCS, a patient is considered diagnosed with the RD when there are at least two instances of any one of the corresponding diagnosis codes in the patient's chart or medical claims data, occurring at least 3 months apart. Two diseases, pheochromocytoma (Pheo) and Charcot Marie Tooth (CMT), did not have specific ICD codes and additional analyses were attempted by adding specific Current Procedural Terminology (CPT) codes to the search criteria (see Results section).

### Percentage of patients

The percentage of patients with a RD was estimated by calculating the number of patients with the disease diagnosis divided by the total number of patients within the HCS database during the specified time period using the source data and HCS approaches summarized in Table 2. For 12 of the 14 RDs that either had specific ICD codes or mapped to 1 or more ICD codes, the 4 HCS databases were searched using the ICD codes listed in Table 1. However, given differences between some of the databases in how patient data is categorized, some customization by system was necessary, including: (1) The NCATS analysis was inclusive of data obtained prior to 2015, and only ICD9 codes were used; and (2) the Eversana database was predominantly organized around billing, and certain non-billable ICD codes were not able to be used in the analyses [for example ICD-9 code 277.0 (Cystic Fibrosis, nonbillable)].

**Table 2:** Aim 1 healthcare system database characteristics and approaches

HCS data source and analytics tools/approach	Number of patients	Geographic area and insurance representation
*NCATS* Database queried using DEVEXI software, a commercially available HIPAA compliant web-based longitudinal health research platform that links de-identified, medical, and dental claims data	~ 9 million patients in entire data warehouse, for which 4.3 million were within 5-year time period (June 5, 2007 to June 6, 2012)* used to estimate disease percentage	State of Florida, ~ 64% live in metropolitan area of at least 1 million people Public 100% (Florida Medicaid/Medicare)
*Eversana* Patient cohorts identified using medical claims data from IBM®Marketscan® Research Database	Inclusive of 195 million patients, in time period 2006–2020, for which only patients first diagnosed with one of the 14 RD and having at least one medical or pharmaceutical claim in from Jan 1, 2013 to Dec 31, 2017 were used to estimate disease percentage.#	Entire U.S Commercial 82%, Public 17%
*Sanford* Custom query of Sanford Epic EHR and associated databases	Inclusive of 1.6 million patients within 5-year time period Jan 1, 2013 to Dec 31, 2017 were used to estimate disease percentage.@	North Dakota, South Dakota, Western Minnesota, Northwest Iowa, Northeast Montana Commercial 42%, Public 58%
*OHSU* Custom queries of OHSU Epic Clarity database and Research Data Warehouse	3.69 million in entire warehouse, ~ 1 million of whom had encounters within 5-year time period Jan 1, 2013 to Dec 31, 2017 and were used to estimate disease percentage	Oregon: 79.07%, Washington: 11.41% Unknown: 5.57% All others: 3.95% *Commercial 46.46%, Public 52.86%, Self-Pay 0.09%, Workers Comp 0.57% *Percentage of visits excluding visits with no listed payer

*NCATS* National Center for Advancing Translational Sciences, *OHSU* Oregon Health and Science University, *EHR* electronic health records

*Most recent 5-year time period data available (ICD-9 coding only because all data prior to 2015)

^#^Prevalence was calculated first by identifying the number of patients first diagnosed with the disease during or prior to that year and having at least one medical or pharmaceutical claim during that year. The percentages of patients with the disease in each year was then calculated by dividing the corresponding number of patients in the database with at least one medical claim during that same year, and finally the percentage of patients with each of the diseases during the 5-year period of 2013–2017 was calculated as the average of the corresponding percentages over the 5 years

^@^The query was further refined by performing a step-wise analysis using increasingly stringent parameters associated with the RD of interest, along with additional data integrity checks, when appropriate (such as ensuring that ICD9 and ICD10 codes matched before and after the transition in 2015), to arrive at the final patient count

The Australia HCS data assessment was performed using the Australian Refined Diagnosis Related Groups (AR-DRG) system, which is an Australian admitted patient classification system that relates the number and type of patients treated in a hospital to the resources required by the hospital, in a clinically meaningful way [25, 26]. AR-DRGs group patients with similar diagnoses requiring similar hospital services. Episodes of admitted hospital acute care are assigned with disease and intervention codes, including Australian Modification ICD-10 (ICD-10-AM) and other coding standards.

The medical literature and public health sources were searched to provide a prevalence estimate comparator for each of the diseases.

### Aim 2: Average direct cost estimates by disease

Direct medical costs were estimated for patients with each of 13 of the 14 RDs identified in Aim 1 using HCS data from 2 of the collaborating institutions NCATS and Eversana. For one disease, CMT, which lacked a specific ICD code, patients were not able to be reliably identified in Aim 1, and this disease was dropped from further analysis. Direct medical costs were estimated using the U.S. dollar amount paid to the HCS that was extracted from the database’s billing records. As per Aim 1, a patient was considered diagnosed with the disease when there were at least two instances of any one of the corresponding diagnoses codes in the patient's medical claims, occurring at least 3 months apart. The first occurrence of the diagnosis satisfying these criteria was defined as the date of diagnosis of the disease for the patient. For the NCATS database, patients were first identified using the RD ICD codes (Aim 1) then direct costs were calculated by disease using billing codes that represent what was paid by the State of Florida’s Medicare/Medicaid program for the time period 2007–2012. For Eversana, direct costs were extracted from the payment information in the IBM® Marketscan® Research Database in years 2006–2020, which includes gross payment made to a provider. For a given RD, the total cost was calculated in each of the years for the set of patients with costs in the database during that given year, independent of the stage of diagnosis (both pre-diagnosis and post-diagnosis).

#### Total cost of care

For NCATS, the total cost of care was calculated by summing the total costs of all visits for each patient in the defined population during the specified time period. For Eversana, the total cost of care for each disease was computed as the sum of cost of care of all patients over all the years.

#### Average cost per patient (PP)

For NCATS, the average cost per patient (PP) in the 5-year time period was derived by calculating the total cost of all visits for each patient in the defined population and the average was then calculated. For Eversana, the total PP cost was calculated in each year for each disease separately by dividing the total cost of care for all patients in that disease cohort in that year by the number of patients in that disease cohort in that year.

#### Per patient per year (PPPY) cost

For NCATS, the PPPY cost was calculated by dividing the average cost over the 5-year time period for each RD population. For Eversana, the PPPY cost for each disease was calculated by averaging the PP over the 15-year time period.

Weight average (wtavg) costs PP for the 13 representative RDs were then calculated using the formula shown in Additional file 2: Figure S1.

#### Control population: average cost of age-matched patients without the rare disease

For NCATS, a control population was created by querying the system for patients that had a general wellness visit within the specified time period. This resulted in patients being pulled with the CPT codes listed as “initial history and examination related to the healthy individual” in adult, adolescent, childhood, and infant age groups (CPT 90750, 90751, 90752, 90754). For Eversana, the average costs for all age-matched patients without the RD within the same HCS database and time period were used as a control using the same methodology.

### Aim 3: Creation of patient journey maps in selected diseases

Using patient-level data in the Eversana (IBM® Marketscan®) database, patient “journey maps” were created, which charted the patient’s clinical course for two diseases, BD CLN2 and CF, for two patients per disease who were identified as having the highest total direct medical costs (Figs. 5, 6). For each patient, key clinical features and major medical milestones, patient characteristics, disease-modifying therapy, and billing costs were extracted from the individual patient records and mapped over the available time period.

## Results

### Aim 1: Estimation of disease prevalence in different HCS databases

**Fig. 1:**
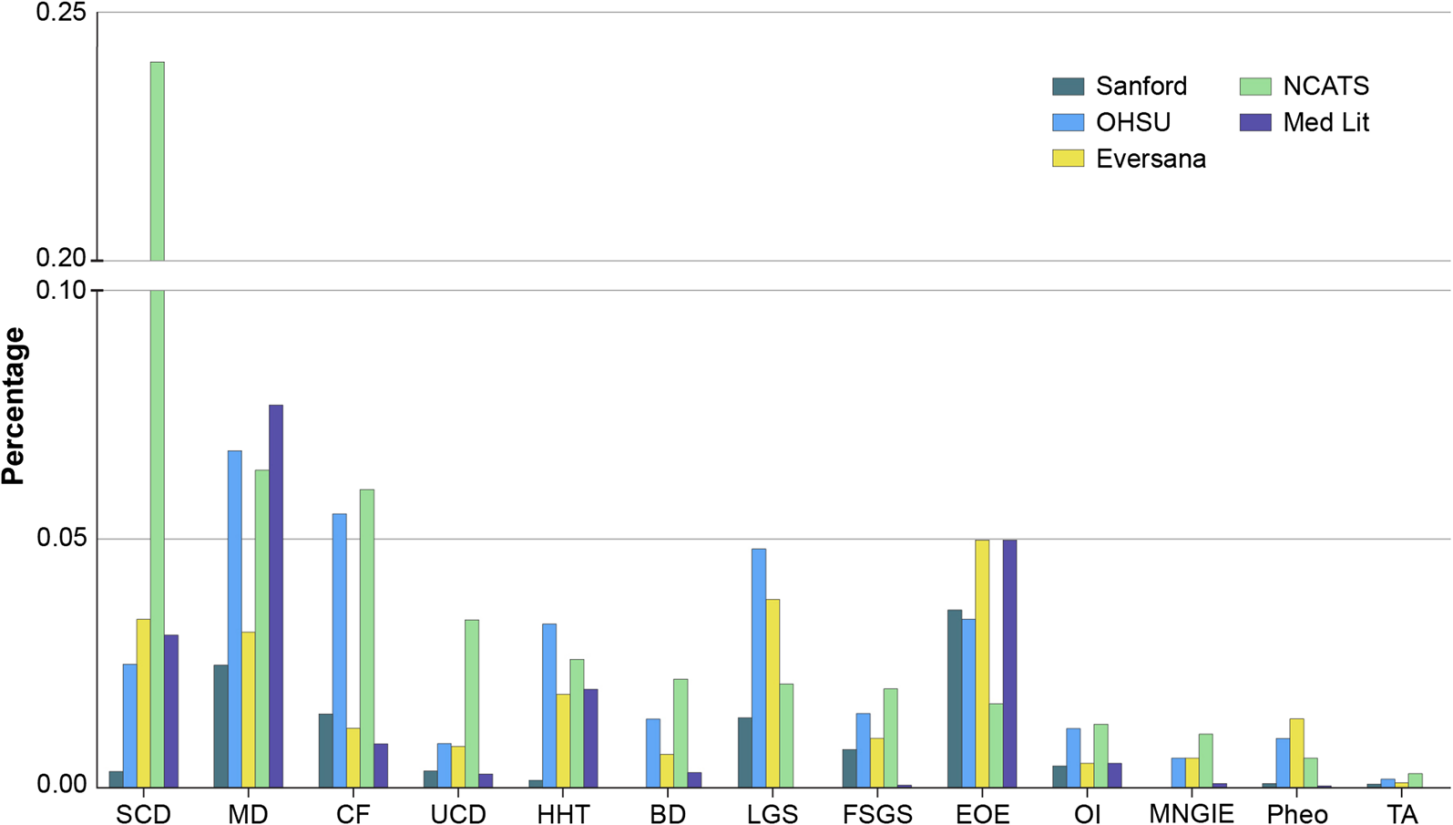
Estimated rare disease percentages by healthcare system database and in the medical literature/published data sources. Percentage of patients with each of the 13 of the 14 representative rare diseases for which a percentage was able to be calculated (excludes CMT) in the 4 healthcare system databases, and disease percentage extrapolated to the US population from the medical literature/public data sources. *SCD* sickle cell disease, *MD* muscular dystrophy, *CF* cystic fibrosis, *HHT* hereditary hemorrhagic teleangiectasia, *BD* Batten disease, *LGS* Lennox Gastaut syndrome, *FSGS* focal segmental glomerulosclerosis, *EOE* eosinophilic esophagitis, *OI* osteogenesis imperfecta, *MNGIE* mitochondrial neurogastrointestinal encephalopathy, *Pheo* pheochromocytoma, *TA* Takayasu’s arteritis, *CMT* Charcot Marie Tooth disease, *NCATS* National Center for Advancing Translational Sciences, *OHSU* Oregon Health and Science University, *Med Lit* medical literature/public data sources

Disease percentage within the HCS databases was estimated by identifying RD patients by ICD codes as a percentage of total patients within the HCS (Fig. 1). The findings show that:

Two of the 14 RDs, Pheo and CMT, do not have specific ICD codes and patients with these diseases were not able to be identified using ICD codes alone. With the aim of more specifically identifying only the patients with these 2 RD of interest, additional analyses were attempted by adding specific CPT codes to the search criteria. For Pheo, which is included under the non-specific ICD code “benign neoplasm of adrenal gland” (ICD-9 227.0) inclusive of several non-related diseases and conditions, the CPT codes for labs more specific to Pheo (e.g., catecholamines) were added to the search criteria as a more sensitive indicator of Pheo vs other benign adrenal tumors (see Table 1). This combined search for Pheo was able to be performed within the 4 remaining HCS databases (NCATS, Eversana, Sanford, OHSU) resulting in a more targeted identification of Pheo patients. A similar strategy for CMT was attempted using the CPT codes thought to be more specific to CMT [e.g., PMP22 (peripheral myelin protein 22)] (see Table 1); however, this approach resulted in 3 of the 4 HCS databases yielding 0 patients, and was not able to provide estimates of the percentage of patients across the different HCS databases. Thus, CMT was dropped from further analysis.

**Fig. 2:**
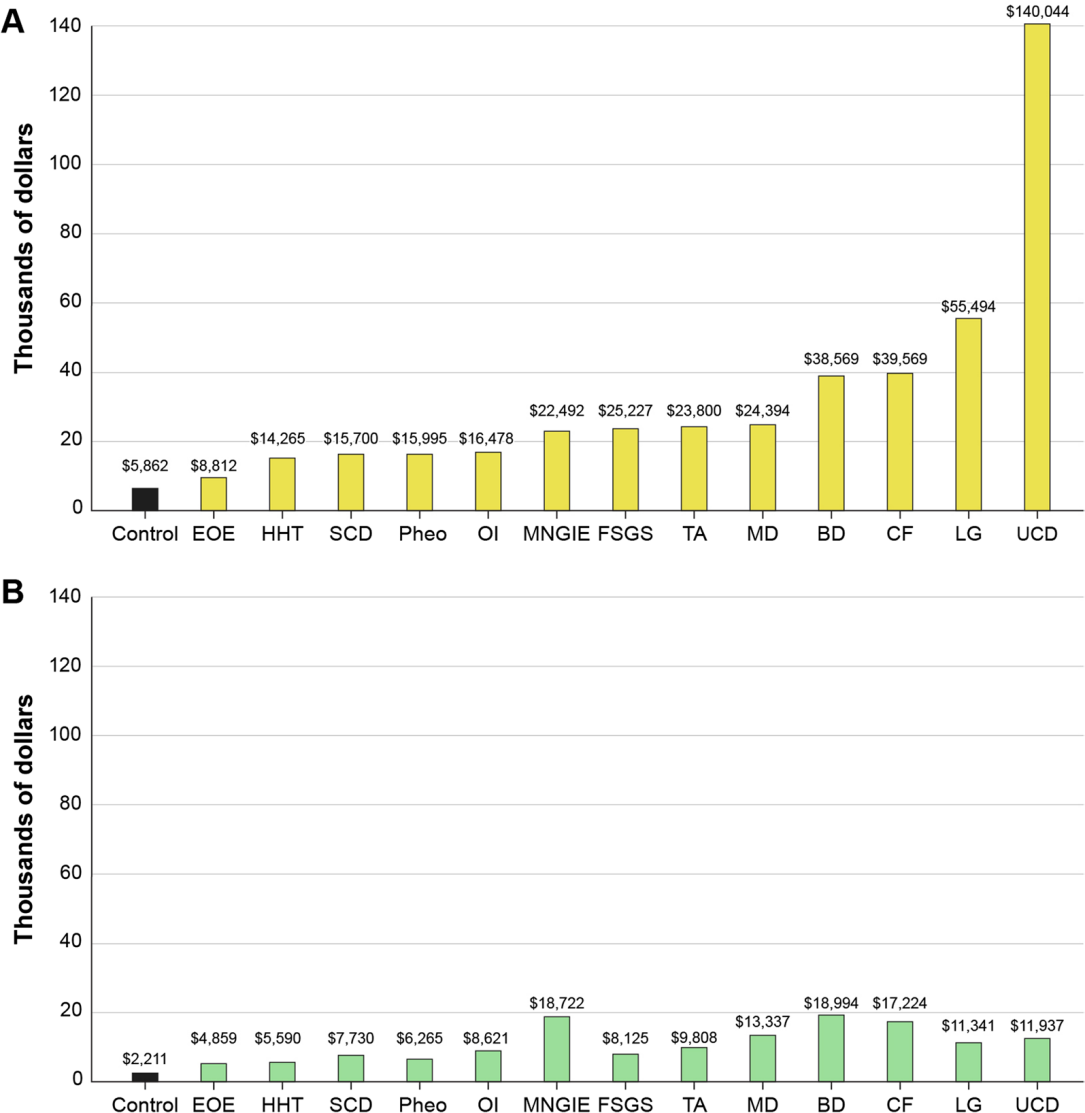
PPPY cost of care of 13 RD versus control. Average per patient per year costs calculated within 2 different healthcare systems databases **A** Eversana and **B** NCATS, versus an age-matched control. *SCD* sickle cell disease, *MD* muscular dystrophy, *CF* cystic fibrosis, *HHT* hereditary hemorrhagic teleangiectasia, *BD* Batten disease, *LGS* Lennox Gastaut syndrome, *FSGS* focal segmental glomerulosclerosis, *EOE* eosinophilic esophagitis, *OI* osteogenesis imperfecta, *MNGIE* mitochondrial neurogastrointestinal encephalopathy, *Pheo* pheochromocytoma, *TA* Takayasu’s arteritis

Second, overall the percentage estimates for the remaining 13 diseases were found to vary widely by HCS (Table 2, Fig. 2). Consistent with the medical literature, Sickle Cell Disease (SCD), Muscular Dystrophy (MD), CF, and Eosinophilic Esophagitis (EoE) had the highest percentages of patients, and Takayasu’s Disease, Pheo, and Mitochondrial NeuroGastroIntestinal Encephalomyopathy (MNGIE) had the lowest. The percentages within a disease were quite variable across the different HCS data analyses, and for many of the diseases, the NCATS analysis showed higher percentages of patients with the selected diseases. These findings may be partially explained by the different populations represented in each of the databases. Many RD, especially genetically-based RD, are known to cluster within certain populations and the variable findings may merely show clustering of populations within certain geographic areas or HCS. For example, many RD are highly debilitating with substantial morbidity that may limit a patient or caregiver’s ability to work or attend school. Thus, RD patients may be disproportionately reliant upon public insurance programs for their healthcare, which may partially explain the higher percentages for some RD in the NCATS findings. The estimates from the medical literature also showed that, in many cases, disease percentages by HCS were not consistent with generally reported literature estimates in that the literature-cited prevalence rates tended to be lower for most of the diseases than the percentages calculated from the HCS databases.

### Aim 2: Average direct cost estimates by disease

#### Cost per patient per year (PPPY)

An evaluation of direct medical costs by disease was estimated independently for the NCATS and Eversana HCS data sources and compared to an age-matched control without the RD. Direct medical costs to payors from HCS billing records were estimated by averaging per patient (PP) cost by disease and total direct costs vs control were estimated by adding the average cost PP by disease over the respective time periods. The results show that average RD costs ranged from 1.5- to 23.9-fold higher versus control (Fig. 2). The Eversana HCS database estimates (Fig. 2a), which were extracted from a mix of commercial and public insurance/payors over an almost 15-year time period (2006–2020), showed per patient per year (PPPY) costs ranged from $8812 to $140,044 for RD patients vs $5862 for the control. The highest PPPY costs for RDs for the Eversana analysis were for Urea Cycle Disorders (UCD), Lennox Gastaut Syndrome (LGS), and BD, and the lowest for EoE, Hereditary Hemorrhagic Telangiectasis (HHT), and SCD. The NCATS estimates (Table 2b), which were extracted from an almost exclusively Medicaid datasource for the 5-year period 2007–2012, PPPY costs ranged from $4859 to 18,994 for RD patients versus $2211 for the control. The highest PPPY costs were for MNGIE, UCD, and MD, and the lowest for EOE, HHT and Pheo. While the NCATS and Eversana cost estimates differed by PPPY and by cost per disease, in every case, the PPPY cost for RD patients exceeded those of the control.

An estimated PPPY cost averaged across the RD was estimated using a weighted average (wtavg). The wtavg for the Eversana analysis was $16,644 for an average RD patient versus $5862 for the control (2.8-fold higher for RD vs control), and for the NCATS analysis was $10,695 for a RD patient versus $2211 for the control (4.8-fold higher).

#### Total cost within time period

**Fig. 3:**
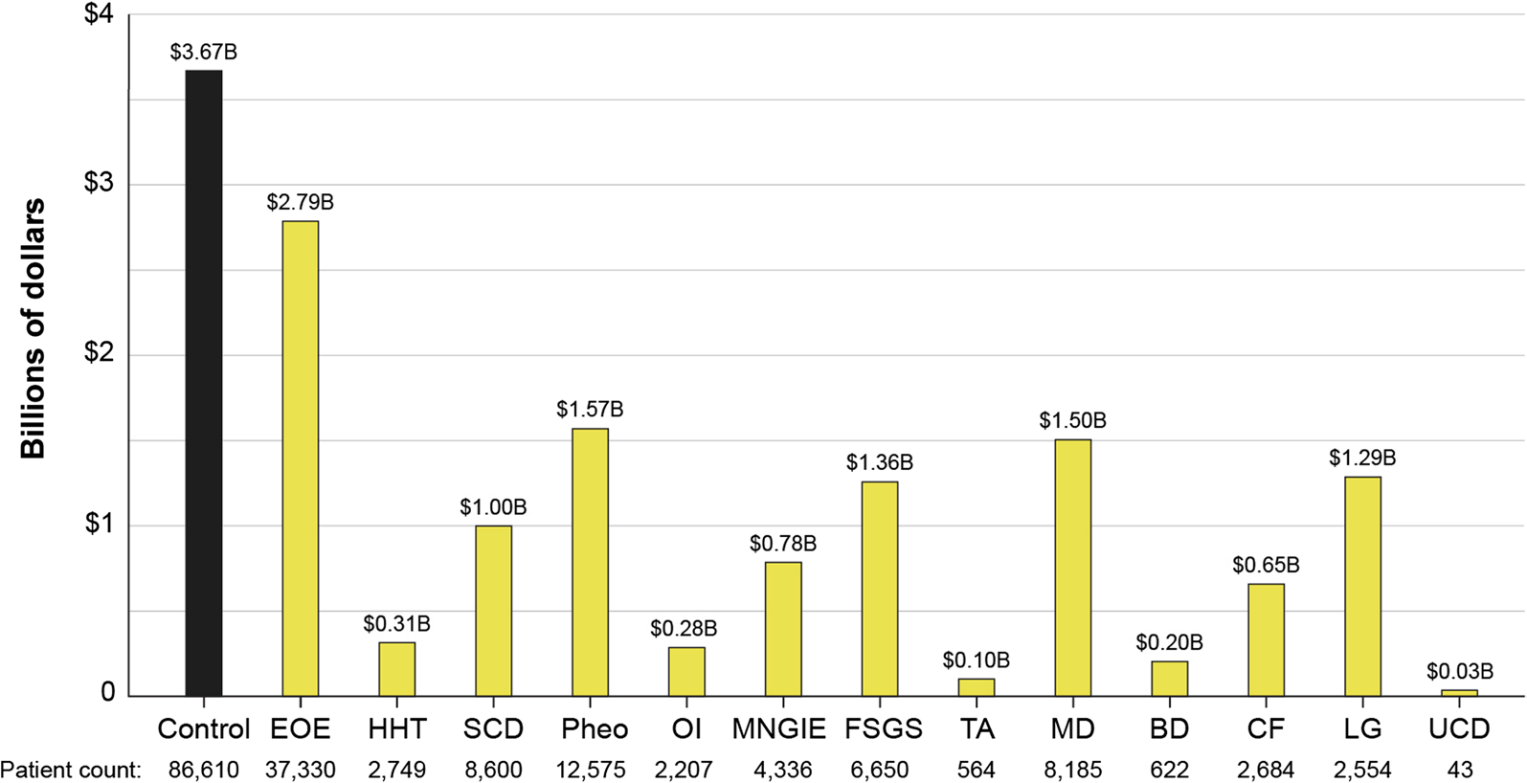
Eversana RD versus control total costs of 13 RD over 15-year time period. Total costs within the 15-year time period 2005–2020 calculated from the Eversana HCS database for 13 representative RD. Costs were calculated by taking the average PPPY cost by disease (Fig. 2a) and multiplying by the number of patients with the disease (Table 3). *SCD* sickle cell disease, *MD* muscular dystrophy, *CF* cystic fibrosis, *HHT* hereditary hemorrhagic teleangiectasia, *BD* Batten disease, *LGS* Lennox Gastaut syndrome, *FSGS* focal segmental glomerulosclerosis, *EOE* eosinophilic esophagitis, *OI* osteogenesis imperfecta, *MNGIE* mitochondrial neurogastrointestinal encephalopathy, *Pheo* pheochromocytoma, *TA* Takayasu’s arteritis

**Fig. 4:**
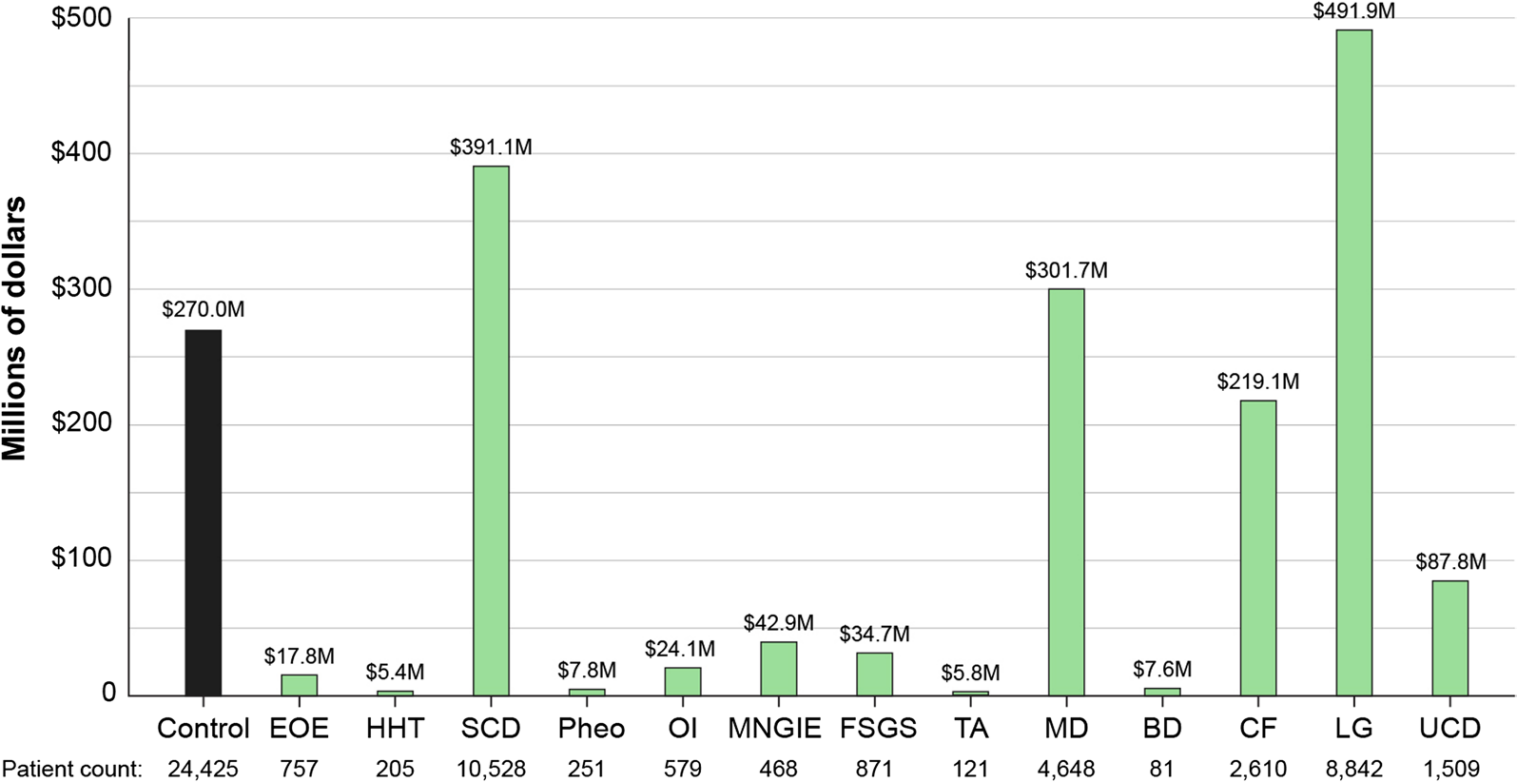
NCATS RD versus control total costs of 13 RD over 5-year time period. Total costs within the 5-year time period 2002–2007 calculated from the NCATSHCS database for 13 representative RD. Costs were calculated by taking the average PPPY cost by disease (Fig. 2b) and multiplying by the number of patients with the disease (Table 3). *SCD* sickle cell disease, *MD* muscular dystrophy, *CF* cystic fibrosis, *HHT* hereditary hemorrhagic teleangiectasia, *BD* Batten disease, *LGS* Lennox Gastaut syndrome, *FSGS* focal segmental glomerulosclerosis, *EOE* eosinophilic esophagitis, *OI* osteogenesis imperfecta, *MNGIE* mitochondrial neurogastrointestinal encephalopathy, *Pheo* pheochromocytoma, *TA* Takayasu’s arteritis

Total costs by RD within the time period, averaged by year, were then calculated by multiplying the number of patients with the disease (or control) by the average cost of the disease (Figs. 3, 4, Table 3). For the Eversana analysis (Fig. 3), the results show that the total costs were higher for the control population and for any individual RD. For NCATS (Fig. 4), there were 3 RD that exceeded the average total costs per year, including LGS, MD and SCD, and with the total costs per disease and control differing from the Eversana data. The reasons for generally lower total costs per disease vs control is likely due to the small number of patients per disease, despite the high average costs PP for RD. The high total costs for the 3 RD in the NCATS analysis vs control are likely due to LGS, MD and SCD being relatively prevalent for a RD, and due to the possible enrichment of patients with RD in a public insurance database.

**Table 3:** Unique patient counts and calculated disease percentages by HCS, and estimates from the medical literature

Total patient count	NCATS		Eversana		Sanford		OHSU		Med lit
	4,333,968		*		1,625,685		1,039,213		N/A
Disease	Unique patient count	Percentage (%)	Unique patient count	Percentage (%)	Unique patient count	Percentage (%)	Unique patient count	Percentage (%)	Estimated prevalence
SCD	10,416	0.2400	38,388	0.0340	55	0.0034%	259	0.0250	0.0308% [38]
MD	2763	0.0640	34,956	0.0313	407	0.0250	709	0.0680	0.0769% [38]
CF	2581	0.0600	13,856	0.0120	246	0.0151	567	0.0550	0.0090% [38]
UCD	1487	0.0340	9423	0.0084	58	0.0036	93	0.0090	0.0029% [39]
HHT	1119	0.0260	21,259	0.0190	28	0.0017	341	0.0330	0.0200% [38]
BD	941	0.0220	7,821	0.0070	3	0.0002	145	0.0140	0.0030% [38]
LGS	917	0.0210	42,837	0.0380	229	0.0141	503	0.0480	0.0001% [38]
FSGS	859	0.0200	11,192	0.0100	128	0.0079	153	0.0150	0.0007% [38]
EOE	755	0.0170	55,055	0.0500	581	0.0357	354	0.0340	0.0500% [38]
OI	573	0.0130	5397	0.0050	73	0.0045	122	0.0120	0.0050% [38]
MNGIE	467	0.0110	7144	0.0060	5	0.0003	66	0.0060	0.0010% [41]
Pheo	250	0.0060	15,521	0.0138	19	0.0012	100	0.0100	0.0005% [40]
TA	117	0.0030	1241	0.0011	14	0.0009	24	0.0020	0.0002% [38]

Unique patient counts by disease extracted from each healthcare system database, and estimated disease percentages within each HCS and for the US population using medical literature/published data sources. Unique patient counts which were used to calculate per patient cost and the total disease cost by disease in Figs. 3, 4

*SCD* sickle cell disease, *MD* muscular dystrophy, *CF* cystic fibrosis, *HHT* hereditary hemorrhagic telangiectasia, *BD* Batten disease, *LGS* Lennox Gastaut syndrome, *FSGS* focal segmental glomerulosclerosis, *EOE* eosinophilic esophagitis, *OI* osteogenesis imperfecta, *MNGIE* mitochondrial neurogastrointestinal encephalopathy, *Pheo* pheochromocytoma, *TA* Takayasu’s arteritis, *CMT* Charcot Marie Tooth disease, *NCATS* National Center for Advancing Translational Sciences, *OHSU* Oregon Health and Science University, *Med Lit* medical literature/public data sources, *N/A* not applicable

*Drawn from ~ 195 million patients in the healthcare system database. Total patient count varied by disease, see Table 1

### Aim 3: Creation of patient journey maps in selected diseases

In order to better understand the disease course leading to diagnosis for RD, with the hopes to identify and diagnose patients with RD sooner after clinical presentation, an exploratory analysis of individual patient journeys were plotted on journey maps, which document key medical events, diagnosis and treatments in 2 RD areas, BD and CF. For this pilot analysis, 2 highest cost patients with each disease were mapped and compared with each other. BD and CF were selected because they have an available disease modifying therapy that allowed for preliminary description of clinical course pre- and post-therapy.

**Fig. 5:**
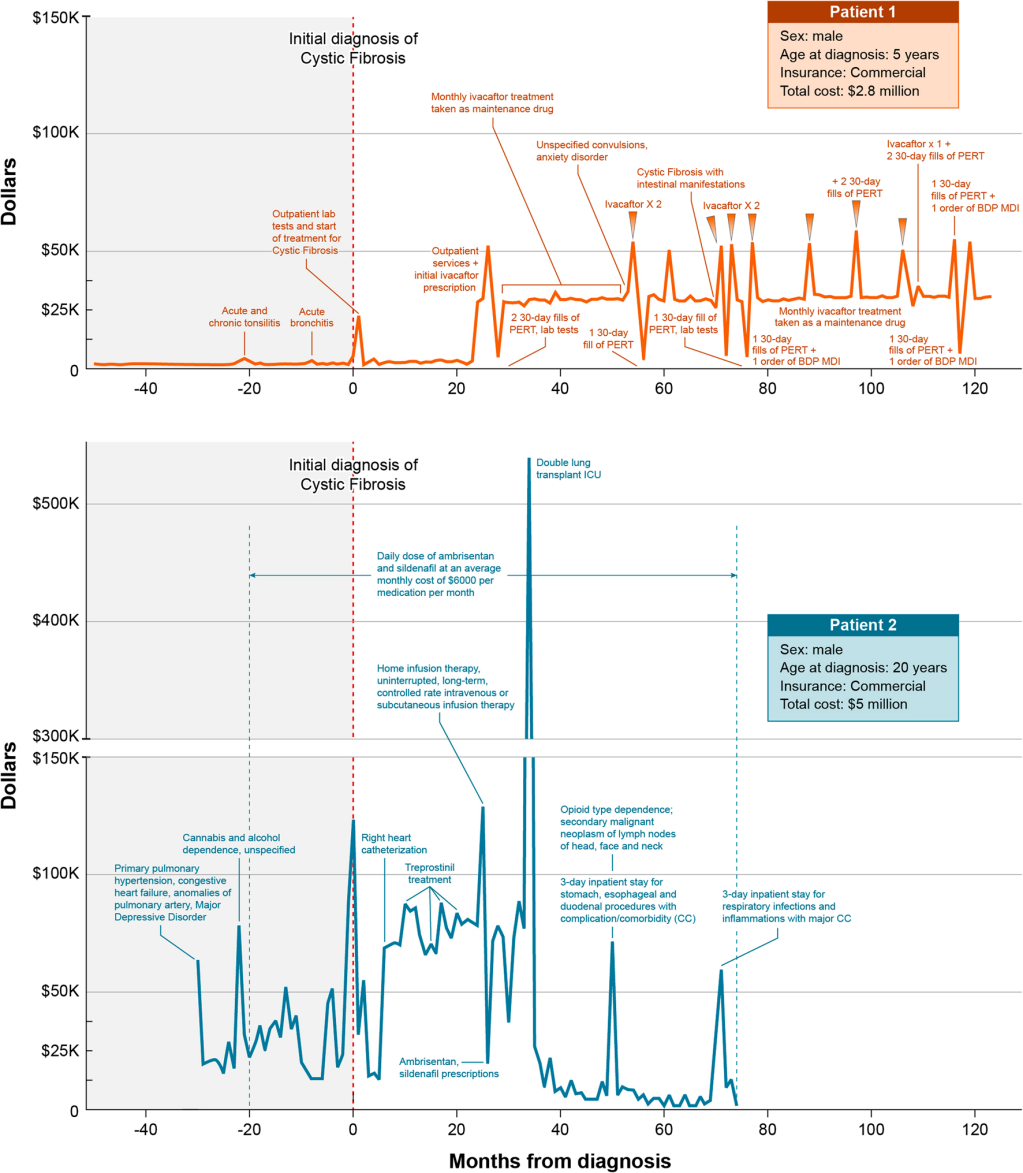
Diagnostic journey maps in 2 high-cost cystic fibrosis patients. *BDP MDI* beclomethasone dipropionate metered dose inhaler, *CC* complication/comorbidity, *ICU* intensive care unit, *PERT* pancreatic enzyme replacement therapy

For CF (Fig. 5), 2 highest costs patients were overlaid, with the date of diagnosis used as time 0 for each patient. The results show the overall clinical course of Patient 1 (red), who experienced 2 upper respiratory tract illnesses approximately 10 and 20 months prior to diagnosis, and was later diagnosed with CF at age 5 years and started on disease modifying therapy (ivacaftor) at approximately 2 years post-diagnosis. Patient 1’s course post-diagnosis shows costs predominantly for prescription drugs, with almost no subsequent clinical events in the post-diagnosis time period. Patient 2 (blue) experienced primary pulmonary hypertension, congestive heart failure, major depressive disorder, and substance abuse disorder clinical events in the approximately 30 months prior to diagnosis, with a CF diagnosis at age 20 years. He subsequently underwent prolonged home infusion therapy and a heart-double lung transplant, accounting for much of the high direct medical costs for this patient.

**Fig. 6:**
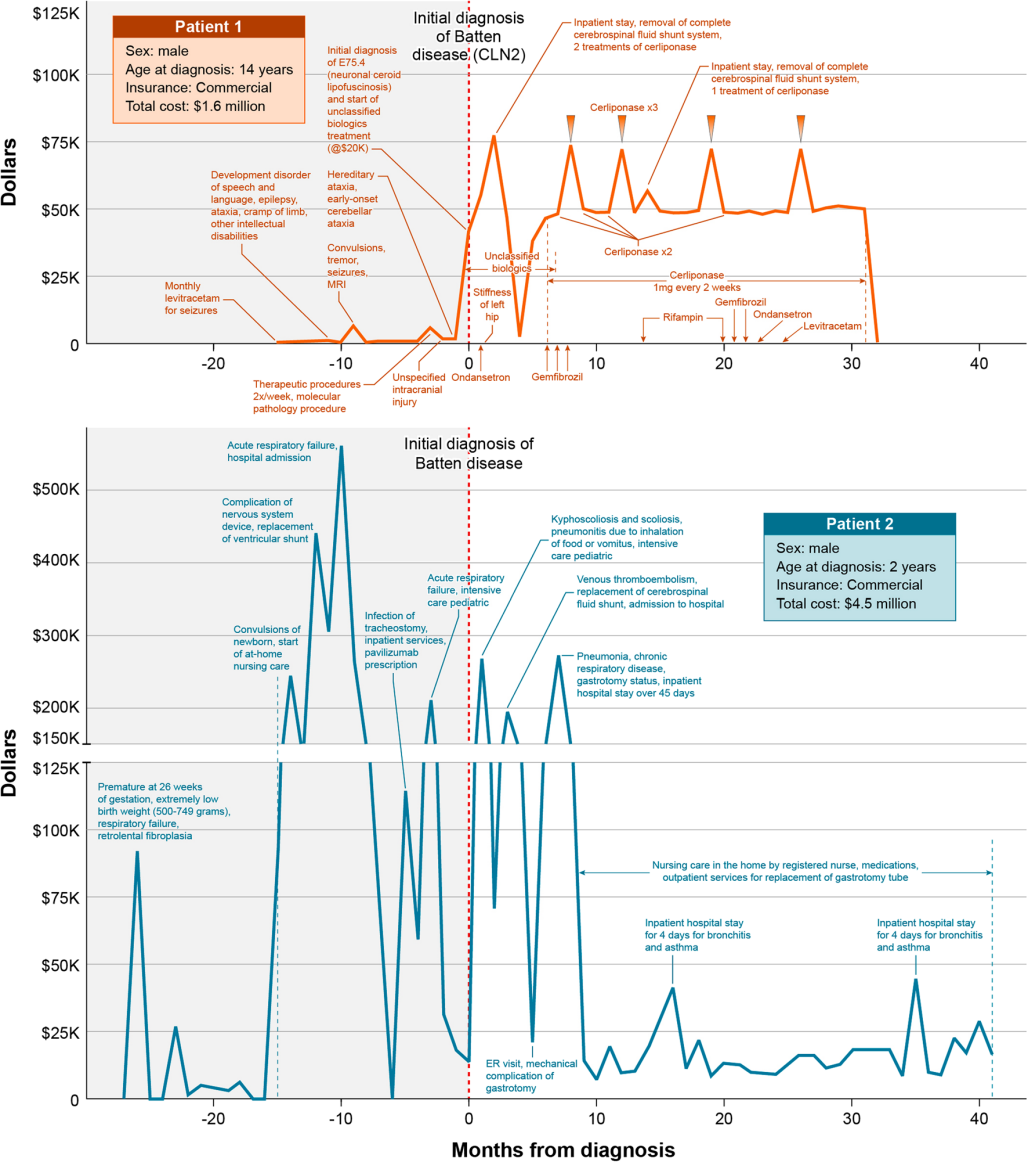
Diagnostic journey maps in 2 high-cost batten disease patients. *CLN2* late infantile neuronal ceroid lipofuscinosis type 2

For BD (Fig. 6), 2 highest costs patients were evaluated, one with CLN2 for which there is an approved disease-modifying therapy, and one unspecified BD patient who did not receive disease-modifying therapy. The results show that pre-diagnosis, Patient 1 (CLN2, red), whose HCS data begins at approximately 12 years of age, had neurodegenerative complications of the disease beginning at the start of his known clinical course, and diagnosis at age 14 years. Disease-modifying therapy (cerliponase) was initiated approximately 4 months after diagnosis, and the patient’s course post-diagnosis reflects costs predominantly for prescription drugs, with two clinical events for BD-related complications (shunt removal) in the post-diagnosis time period. Patient 2 (BD, blue) had premature birth, numerous ICU and other hospitalizations for convulsions, respiratory failure, nervous system procedures, and other complications of BD, with subsequent diagnosis at age 2 years, and post-diagnosis events, including ICU and hospitalizations relating to neurodegenerative and respiratory complications of the disease, and eventual transition to home nursing care.

## Discussion

In this pilot study, we explored the feasibility of quantifying the number of RD patients within different HCS and the direct medical costs for their care, and performed a preliminary analysis of the diagnostic journey for individual RD patients. The results are notable for three major findings.

First, estimating RD percentages within and across different databases and HCS using straight-forward ICD code search strategies is not able to provide reliable or consistent RD patient identification or disease percentage estimates. We saw wide variability in percentage estimates for 14 representative RD, which may, in part, be due to differences in patient populations within the different HCS, the different types of HCS, and the type of data being queried (EHR data vs medical claims data). Given that many RD are genetic, clustering of patients in geographic areas or different payor systems with specialized expertise is not unexpected; however, in preliminary analysis of the diagnostic journey, and as reported by others, [12, 13] we know that many RD patients undergo prolonged periods of time where they are undiagnosed or misdiagnosed, which also may contribute to small percentages and variability across HCS. Furthermore, the lack of infrastructure for sharing RD knowledge and tools for diagnosis in HCS could lead to disparities in diagnostic rate and time to diagnosis. For the 2 RD in our sample that did not have a specific ICD codes (Pheo, CMT), identifying patients with these conditions was even more difficult. Pheo patients were relatively consistently identified across the different HCS by developing customized search criteria, in this case using specific CPT codes, but CMT patients could not be reliably identified using a similar approach. Given that at least half of RD do not currently map to a specific ICD code, consistently and reliably quantifying the estimated 25–30 million RD patients in the US with the thousands of different RD is a daunting task that would require individualized and computable phenotyping criteria for most RD.

Identifying and quantifying RD patients internationally was shown to be even more difficult. Different countries use different approaches for patient classification and payment, which may not be readily applied in other HCS, and our attempts to combine the AR-DRG system into the study were unsuccessful. Interoperability and data/knowledge sharing are crucial to improve the ability for HCS to diagnose and care for patients. RD, being rare, require this knowledge and data from around the world be utilized in local HCS; our attempts to identify and profile RD patients in Australia highlight this persisting need. Many open science initiatives exist to overcome these issues; however, coding systems, classification strategies, and tools for sharing RD case information have yet to be implemented in most HCS. Further, important data and knowledge are needed directly from patients, such as from registries, natural history studies and biobanks, however, these important datasources that could provide this knowledge [27] have not to date been integrated into HCS. A call to action to make data and knowledge openly shareable and interoperable into HCS was recently published [28].

Second, RD direct medical costs are high, with RD average PPPY costs estimated to be approximately three to fivefold higher than age-matched controls. While there were differences in total direct costs PP depending on different payors HCS used, the PP costs were still consistently higher across the RD in this sample when compared to non-RD patients. This result is not unexpected—high direct medical costs and healthcare utilization are surrogates for poor health. Patients with complex conditions and serious illnesses, regardless of type or rarity, are generally heavily reliant upon healthcare services to sustain life and relieve pain and suffering with resultant high costs to patients and their families, HCS, and society writ large. Most RD are genetic disorders that interrupt or affect fundamental biological processes (e.g., enzyme deficiencies), are overwhelmingly serious and life-threatening conditions, often affecting more than one organ system, which result in substantial impacts to the patient’s overall health and activities of daily living. Unlike most other illnesses however, RD disproportionately (but not exclusively) affect younger patients—children, adolescents, and young adults—with impacts, on average, showing substantially higher costs versus age-matched non-RD patients.

We additionally note that the total cost of an individual RD was generally lower than for the control overall. Given the fragmentation of small numbers of RD patients across thousands of different disorders and despite the relatively high PP costs per RD disease, many RD are likely to have a relatively low total cost (PP cost times the number of patients) that may not stand out within HCS, and thus, not call sufficient attention to the seriousness and high clinical needs for many RD.

Third, preliminary assessment of high-cost RD patients with two RD (CF, BD) showed that these patients had long (ranging for ~ 1.5 to 20 years) diagnostic journeys after first clinical presentation prior to receiving a definitive diagnosis, which for 3 of the 4 patients described resulted in the occurrence of irreversible complications of the disease and ongoing high costs and HCS utilization related to disease progression.

Mapping of the clinical course also showed that there is potential for identifying and diagnosing suspected RD patients sooner. These patients showed recurrent engagement with the HCS, persistent and progressive symptoms often falling into more general “basket” terms (e.g., convulsions, developmental delay, recurrent infections), and high utilization relative to age-matched controls. These patterns could be leveraged to escalate patients for definitive diagnosis and intervention sooner in order to slow disease progression or avoid catastrophic presentations and hospital admissions (e.g., organ transplant, ICU stays) [29]. We saw candidate diagnoses within the problem lists, and although often found in clinical notes, they may not be documented as diagnoses until later time points. The administration of disease-modifying treatments showed changes in clinical course in the two patients in this study. While high-costs continued post-diagnosis and treatment administration, the costs for the treated patients almost entirely clustered into the costs for outpatient treatment administration vs continuing hospital care for the patients without a disease-modifying therapy. This signal in individual patients shows hope for earlier diagnosis and intervention, where available, potentially offering beneficial effects and altering the clinical course in some RD.

### Study limitations

There were several limitations to this study. The study was intended to be a pilot/exploratory study to assess the feasibility of identifying and quantifying costs and utilization in RD in a select sample of 14 RD. Although the sample of RD was chosen to reflect the diversity of RD, with widely varying presentations, clinical course, age and populations affected in this sample, the 14 RD admittedly represent only a small sample of the estimated 7–10,000 different RD and 25–30 million patients in the US with RD, and it is not known if these RD are truly representative of the RD population generally. This study was also intended as a preliminary feasibility pilot to begin to address the large problem of identifying, describing and quantifying RD patient data within the US healthcare system, which could then be used to answer larger research questions currently beyond the scope of the IDeaS analyses, such as relationships between costs and cost savings, patient outcomes and disease rarity. However, we see the current analyses as important first steps in what is intended to be an iterative process of developing methodologies that can progressively and deliberately address these larger research goals over time. Additionally, the widely varying percentages of these diseases in different HCS and versus commonly cited literature sources makes it difficult to understand the true prevalence of RD in HCS in the US. The information sources presented additional limitations. Data included in the EHR, but not placed in structured data fields is not available for simple extraction and limits the ability to identify RD diagnoses. While this may occur with both rare and common disease diagnoses, it disproportionately affects RD because only about half of RD can be mapped to a more specific ICD code or cluster, as well as the prolonged timelines between symptom/disease onset and accurate diagnosis and coding of RD patients that make them especially difficult to identify within HCS. Additionally, US patients frequently change their HCS plans and lack of continuity of data from one EHR or HCS to another makes it difficult to identify original diagnosis dates or sentinel signs/terms that may facilitate RD recognition [30]. Thus, taken together, our study suggests that RD patients have long diagnostic journeys compounded by lack of HCS continuity, and tend to be classified under broader non-specific terms, at least early on in their disease course, resulting in percentage estimates that are likely to be underestimates of their true prevalence and impact of RD on HCS.

Direct costs are also based on the costs to payors, which are known to differ substantially by type of insurance (or no insurance) for individual patients. PP and total costs in the 2 HCS presented in this study varied widely, and likely reflect differences in the payor status (e.g., commercial vs public) in the two HCS. However, in either case, RD costs PP were still notably higher than matched control. Direct medical costs also only account for a portion of total medical costs on patients, families, and HCS. We were not able to assess out-of-pocket costs and indirect costs (such as social and support services) that patients and societies incur for RD patient care and treatment.

## Conclusions

Overall, these preliminary findings suggest several major considerations for RD that should form the basis for additional study.

- RD patients are likely to be under-recognized and under-estimated in HCS databases and in cost estimates for their medical care. This under-estimation results in the lack of recognition of the true scope of the public health impact of RD on HCS, as well as the vast unmet and ongoing medical needs for RD patients.
- PP costs on average in this study were around three- to fivefold higher than a matched control; gross extrapolation of this average costs estimate in a large HCS database (Eversana, estimated at approximately ~ $17 K per RD patient per year vs ~ $6 K for the control) for an estimated 25 million RD patients in US would result in total yearly direct medical costs for RD in the range of $400 billion per year, making the cost burden similar to other high-cost diseases, such as cancer [31] and heart failure, [32] and exceeding those of Alzheimer’s disease [33]. Additionally, the large variance in the cost of care of patients with the same RD could be attributed to different reasons—using HCS and insurance claims databases to stratify patient cohorts within a given RD to surface diagnostic, therapeutic, and utilization patterns will be valuable in the quest to better understand disease course and uncover ideal disease management interventions.
- Machine-assisted strategies for early identification and diagnosis of likely RD patients may be feasible. Journey maps in selected RD patients revealed potential characteristics, such as young age, high utilization, recurrent hospitalizations and severe clinical presentations, that may assist with early identification and escalation for definitive diagnosis. Genetic diagnosis as part of the early diagnosis strategy has been shown to be beneficial in other analyses, and importantly, impact clinical course and patient management, especially if implemented earlier [34–37].

Thus, we conclude that the results from this small pilot study of RD impact on HCS show that the 14 RD included in this pilot have high medical burdens to patients and HCS, likely in a similar range to burdens experienced by patients with other serious diseases, such as cancer, heart failure and Alzheimer’s disease; however, these results will need to be confirmed in a larger cohort of RD. This suggests that RD represent a major impact to public health, have high unmet medical needs, and that there is an urgent and considerable need for earlier and accurate RD diagnosis and intervention to address medical management for RD patients that is further supported by similar high-cost burden results seen in two other recent cost-burden studies [21, 22].

Finally, with the information and data gathered from this small pilot study, we have sought to bring attention to key considerations (such as limitations in coding) that have been recognized for many years in the RD community that continue to limit our ability to better understand RD and their impacts on patients and the public health. This is an important line of inquiry and we hope that efforts such as this study, will begin to open new areas of research that can improve our ability to identify RD patients more accurately, and assess and mitigate the impacts (utilization and cost) of RDs by leveraging available HCS data.

## Supplementary Information

**Additional file 1**. **Table S1**: ICD and CPT Codes and Descriptions.

**Additional file 2**.** Figure S1**. Weighted Average Cost Formula Weighted average (wtavg) costs were calculated by taking the sum of the number of patients in each RD cohort (#ptRD1-13) and dividing it by the sum of all RD patient cohorts (sum pt), then multiplying by the sum of the PPPY costs of all RD patient cohorts (sumPPPY). Then the individual RD weighted averages were combined to create a weighted average for our total 13 RD population (wtavg RDpop) Abbreviations: WTavg, weighted average, RDPOP, total population for 13 representative RD, #pt, number of patients, sumPPPY, sum of the PPPY costs of all RD patient cohorts.

## Data Availability

The datasets are not publicly available because the information was extracted from healthcare systems databases, but pooled/summary datasets are available from the corresponding author on reasonable request.

## Abbreviations

AKA: Also known as
AR-DRG: Australian Refined Diagnosis Related Groups
BD: Batten disease
BDP MDI: Beclomethasone dipropionate metered dose inhaler
CC: Complication/comorbidity
CF: Cystic fibrosis
CLN2: Late infantile neuronal ceroid lipofuscinosis type 2
CMT: Charcot Marie Tooth disease
CPT: Current Procedural Terminology
EEG: Electroencephalogram
EHR: Electronic health record
EOE: Eosinophilic esophagitis
FSGS: Focal segmental glomerulosclerosis
GI: Gastrointestinal
HHT: Hereditary hemorrhagic telangiectasis
HCS: Healthcare system
ICD: International Classification of Disease
LGS: Lennox Gastaut syndrome
MD: Muscular dystrophy
MNGIE: Mitochondrial neurogastrointestinal encephalopathy
NCATS: National Center for Advancing Translational Sciences
NCL: Neuronal ceroid lipofuscinosis
NIH: National Institutes of Health
OI: Osteogenesis imperfecta
OHSU: Oregon Health and Science University
ORDR: Office of Rare Diseases Research
PERT: Pancreatic enzyme replacement therapy
Pheo: Pheochromocytoma
PP: Per patient
PPPY: Per patient per year
RD: Rare diseases
SCD: Sickle cell disease
TA: Takayasu’s arteritis
UCD: Urea cycle disorders
US: United States
Wtavg: Weighted average
