# Supplementary material for: The IDeaS initiative: pilot study to assess the impact of rare diseases on patients and healthcare systems
Source: Orphanet J Rare Dis. 2021 Oct 22;16:429. doi: 10.1186/s13023-021-02061-3 (PMC8532301; doi:10.1186/s13023-021-02061-3)
Supplement: Supplementary file 1 — Additional file 1. Table S1: ICD and CPT Codes and Descriptions. [file 13023_2021_2061_MOESM1_ESM.docx]

| Disease/Disease Area | ICD Version or CPT | ICD/CPT Code | ICD/CPT Description |
| --- | --- | --- | --- |
| **Batten Disease (BD)** | **ICD-10** | **E75.4** | Neuronal ceroid lipofuscinosis |
|  | ICD-10 | G11.1 | Early-onset cerebellar ataxia |
|  | ICD-9 | 334.3 | Other cerebellar ataxia |
|  | ICD-9 | 330.1 | Cerebral lipidoses |
| **Charcot Marie Tooth (CMT)** | ICD-9 | 356.1 | Peroneal muscular atrophy- |
|  | ICD-10 | G60.0 | Hereditary motor and sensory neuropathy |
|  | CPT | 81324 | PMP22 (peripheral myelin protein 22) (eg; Charcot-Marie-Tooth; hereditary neuropathy with liability to pressure palsies) gene analysis; duplication/deletion analysis |
|  | CPT | 81326 | PMP22 (peripheral myelin protein 22) (eg; Charcot-Marie-Tooth; hereditary neuropathy with liability to pressure palsies) gene analysis; known familial variant |
|  | CPT | 81325 | PMP22 (peripheral myelin protein 22) (eg; Charcot-Marie-Tooth; hereditary neuropathy with liability to pressure palsies) gene analysis; full sequence analysis |
| **Cystic Fibrosis (CF)** | ICD-10 | E84.0 | Cystic fibrosis with pulmonary manifestations |
|  | ICD-10 | E84.19 | Cystic fibrosis with other intestinal manifestations |
|  | ICD_10 | E84.8 | Cystic fibrosis with other manifestations |
|  | ICD-10 | E84.9 | Cystic fibrosis, unspecified |
|  | ICD-10 | E84.11 | Meconium ileus in cystic fibrosis |
|  | ICD-9 | 277.00 | Cystic fibrosis |
|  | ICD-9 | 277.02 | Cystic fibrosis with pulmonary manifestations |
|  | ICD-9 | 277.03 | Cystic fibrosis with gastrointestinal manifestations |
|  | ICD-9 | 277.09 | Cystic fibrosis with other manifestations |
|  | ICD-9 | 277.01 | Cystic fibrosis with meconium ileus |
|  | ICD-9 | 277.0 | Cystic Fibrosis, nonbillable |
| **Eosinophilic Esophagitis (EOE)** | ICD-10 | K20.0 | Eosinophilic esophagitis |
|  | ICD-9 | 530.13 | Eosinophilic esophagitis |
| **Focal and Segmental Glomerulosclerosis (FSGS)** | ICD-9 | 581.0 | Nephrotic syndrome with lesion of proliferative glomerulonephritis |
|  | ICD-9 | 581.1 | Nephrotic syndrome with lesion of membranous glomerulonephritis |
|  | ICD-9 | 581.2 | Nephrotic syndrome with lesion of membranoproliferative glomerulonephritis |
|  | ICD-9 | 581.3 | Nephrotic syndrome with lesion of minimal change glomerulonephritis |
|  | ICD-10 | N04.0 | Nephrotic syndrome with minor glomerular abnormality |
|  | ICD-10 | N04.1 | Nephrotic syndrome w focal and segmental glomerular lesions |
|  | ICD-10 | N04.2 | Nephrotic syndrome w diffuse membranous glomerulonephritis |
|  | ICD-10 | N04.7 | Nephrotic syndrome w diffuse crescentic glomerulonephritis |
|  | ICD-10 | N03.1 | Chronic nephritic syndrome with focal and segmental glomerular lesions |
| **Hereditary Hemorrhagic Telangiectasia (HHT)** | ICD-9 | 448.0 | Hereditary hemorrhagic telangiectasia |
|  | ICD-9 | 448.9 | other, unspecified capillary diseases |
|  | ICD-10 | I78.0 | Hereditary hemorrhagic telangiectasia |
| **Lennox Gastaut Syndrome (LGS)** | ICD-10 | G40.812 | Lennox-Gastaut syndrome, not intractable, w/o stat epi |
|  | ICD-10 | G40.811 | Lennox-Gastaut syndrome, not intractable, w stat epi |
|  | ICD-10 | G40.813 | Lennox-Gastaut syndrome, intractable, w stat epi |
|  | ICD-10 | G40.814 | Lennox-Gastaut syndrome, intractable, w/o status epilepticus |
|  | ICD-9 | 345.01 | generalized nonconvulsive epilepsy, with intractable epilepsy |
|  | ICD-9 | 345.00 | Generalized nonconvulsive epilepsy, without mention of intractable epilepsy |
|  | ICD-9 | 345.0 | Generalized nonconvulsive epilepsy |
|  | ICD-9 | 345.1 | Generalized convulsive epilepsy |
| **Mitochondrial neurogastrointestinal encephalopathy (MNGIE)** | ICD-9 | 277.87 | Disorders of mitochondrial metabolism |
|  | ICD-10 | E88.49 | Other mitochondrial metabolism disorders |
| **Muscular Dystrophy (MD)** | ICD-10 | G71.0 | Muscular dystrophy |
|  | ICD-10 | G71.01 | Duchenne or Becker muscular dystrophy |
|  | ICD-10 | G71.00 | Muscular dystrophy, unspecified |
|  | ICD-10 | G71.02 | Facioscapulohumeral muscular dystrophy |
|  | ICD-10 | G71.09 | Other specified muscular dystrophies |
|  | ICD-10 | G71.11 | Myotonic muscular dystrophy |
|  | ICD-9 | 359.21 | Myotonic muscular dystrophy |
|  | ICD-9 | 359.22 | Myotonia congenita |
|  | ICD-9 | 359.23 | Myotonic chondrodystrophy |
|  | ICD-9 | 359.1 | Hereditary progressive muscular dystrophy |
|  | ICD-9 | 359.0 | Congenital hereditary muscular dystrophy |
|  | ICD-9 | 359.9 | myopathy unspecified |
|  | ICD-9 | 359.29 | Other specified myotonic disorder |
| **Osteogenesis Imperfecta (OI)** | ICD-10 | Q78.0 | Osteogenesis imperfecta |
|  | ICD-9 | 756.51 | Osteogenesis imperfecta |
| **Pheochromocytoma (Pheo)** | CPT | 82384 | Catecholamines; fractionated |
|  | CPT | 82382 | Catecholamines; total urine |
|  | CPT | 83835 | Metanephrines |
|  | FOLLOWED BY | | |
|  | ICD-10 | C74.10 | Malignant neoplasm of medulla of unspecified adrenal gland |
|  | ICD-10 | C96.20 | malignant mast cell neoplasm, unspecified |
|  | ICD-10 | C96.29 | other malignant mast cell neoplasm |
|  | ICD-10 | D35.00 | Benign neoplasm of adrenal gland |
|  | ICD-9 | 227.0 | Benign neoplasm of adrenal gland |
| **Sickle Cell Disease (SCD)** | ICD-9 | 282.60 | Sickle-cell disease |
|  | ICD-9 | 282.61 | Hb-SS disease without crisis |
|  | ICD-9 | 282.62 | Hb-SS disease with crisis |
|  | ICD-9 | 282.63 | Sickle-cell/Hb-C disease without crisis |
|  | ICD-9 | 282.64 | Sickle-cell/Hb-C disease with crisis |
|  | ICD-9 | 282.68 | Other sickle-cell disease without crisis |
|  | ICD-9 | 282.69 | Other sickle-cell disease with crisis |
|  | ICD-9 | 282.6 | Sickle-cell disease |
|  | ICD-9 | 282.41 | Sickle-cell thalassemia without crisis |
|  | ICD-9 | 282.42 | Sickle-cell thalassemia with crisis |
|  | ICD-10 | D57.00 | Hb-SS disease with crisis, unspecified |
|  | ICD-10 | D57.01 | Hb-SS disease with acute chest syndrome |
|  | ICD-10 | D57.02 | Hb-SS disease with splenic sequestration |
|  | ICD-10 | D57.1 | Sickle-cell disease without crisis |
|  | ICD-10 | D57.20 | Sickle-cell/Hb-C disease without crisis |
|  | ICD-10 | D57.211 | Sickle-cell/Hb-C disease with acute chest syndrome |
|  | ICD-10 | D57.212 | Sickle-cell/Hb-C disease with splenic sequestration |
|  | ICD-10 | D57.219 | Sickle-cell/Hb-C disease with crisis, unspecified |
|  | ICD-10 | D57.40 | Sickle-cell thalassemia without crisis |
|  | ICD-10 | D57.411 | Sickle-cell thalassemia with acute chest syndrome |
|  | ICD-10 | D57.412 | Sickle-cell thalassemia with splenic sequestration |
|  | ICD-10 | D57.419 | Sickle-cell thalassemia with crisis, unspecified |
|  | ICD-10 | D57.80 | Other sickle-cell disorders without crisis |
|  | ICD-10 | D57.811 | Other sickle-cell disorders with acute chest syndrome |
|  | ICD-10 | D57.812 | Other sickle-cell disorders with splenic sequestration |
|  | ICD-10 | D57.819 | Other sickle-cell disorders with crisis, unspecified |
| **Takayasu’s Arteritis (TA)** | ICD-9 | 446.7 | Takayasu's disease |
|  | ICD-10 | M31.4 | Aortic arch syndrome [Takayasu] |
| **Urea Cycle Disorder (UCD)** | ICD-9 | 270.6 | disorders of urea cycle metabolism |
|  | ICD-10 | E72.2 | disorders of the urea cycle metabolism |
|  | ICD-10 | E72.20 | Disorder of urea cycle metabolism, unspecified |
|  | ICD-10 | E72.21 | Argininemia |
|  | ICD-10 | E72.22 | Arginosuccinic aciduria |
|  | ICD-10 | E72.23 | Citrullinemia |
|  | ICD-10 | E72.29 | Other disorders of urea cycle metabolism |
|  | ICD-10 | E72.4 | Disorders of ornithine metabolism |
