# Supplementary material for: The IDeaS initiative: pilot study to assess the impact of rare diseases on patients and healthcare systems
Source: Orphanet J Rare Dis. 2021 Oct 22;16:429. doi: 10.1186/s13023-021-02061-3 (PMC8532301; doi:10.1186/s13023-021-02061-3)
Supplement: Supplementary file 2 — Additional file 2. Figure S1. Weighted Average Cost Formula Weighted average (wtavg) costs were calculated by taking the sum of the number of patients in each RD cohort (#ptRD1-13) and dividing it by the sum of all RD patient cohorts (sum pt), then multiplying by the sum of the PPPY costs of all RD patient cohorts (sumPPPY). Then the individual RD weighted averages were combined to create a weighted average for our total 13 RD population (wtavg RDpop) Abbreviations: WTavg, weighted average, RDPOP, total population for 13 representative RD, #pt, number of patients, sumPPPY, sum of the PPPY costs of all RD patient cohorts. [file 13023_2021_2061_MOESM2_ESM.docx]

**Figure S1. Weighted Average Cost Formula**

Wtavg =
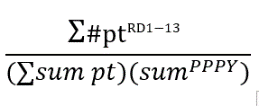


Wtavg^RDpop^ = ∑Wtavg

Weighted average (wtavg) costs were calculated by taking the sum of the number of patients in each RD cohort (#pt^RD1-13^) and dividing it by the sum of all RD patient cohorts (sum pt), then multiplying by the sum of the PPPY costs of all RD patient cohorts (sum^PPPY^). Then the individual RD weighted averages were combined to create a weighted average for our total 13 RD population (wtavg ^RDpop^)

Abbreviations: WTavg, weighted average, RD^POP^, total population for 13 representative RD, #pt, number of patients, sum^PPPY^, sum of the PPPY costs of all RD patient cohorts
